# Rethinking Disease Preparedness: Incertitude and the Politics of Knowledge

**DOI:** 10.1080/09581596.2021.1885628

**Published:** 2021-02-24

**Authors:** Melissa Leach, Hayley MacGregor, Santiago Ripoll, Ian Scoones, Annie Wilkinson

**Affiliations:** Institute of Development Studies, University of Sussex, Brighton, UK

**Keywords:** Preparedness, incertitude, risk, knowledge

## Abstract

This paper argues for a rethinking of disease preparedness that puts incertitude and the politics of knowledge at the centre. Through examining the experiences of Ebola, Nipah, cholera and COVID-19 across multiple settings, the limitations of current approaches are highlighted. Conventional approaches assume a controllable, predictable future, which is responded to by a range of standard interventions. Such emergency preparedness planning approaches assume risk – where future outcomes can be predicted – and fail to address uncertainty, ambiguity and ignorance – where outcomes or their probabilities are unknown. Through examining the experiences of outbreak planning and response across the four cases, the paper argues for an approach that highlights the politics of knowledge, the constructions of time and space, the requirements for institutions and administrations and the challenges of ethics and justice. Embracing incertitude in disease preparedness responses therefore means making contextual social, political and cultural dimensions central.

## Introduction

The COVID-19 pandemic is the latest disease outbreak to affect the world, unfolding across multiple countries with devastating impacts on health, economies and societies. In 2016, following the West African Ebola outbreak, the Commission on a Global Health Risk Framework (GHRF) for the Future argued that “While it is impossible to produce precise estimates for the probability and potential impact of pandemics, it is not difficult to demonstrate a compelling case for greater investment” (GHRF Commission 2016: 17).

Since 2016 there have been such investments and a host of global meetings and reports on epidemics that have focused on centralised planning, modelling and prediction capacity, surveillance systems, big data technologies, rapid response teams, drug and vaccine research and development, stockpiling and purchase arrangements and health system strengthening. In 2017, WHO established its guidelines for health emergency preparedness (WHO 2017a), extending the ambit of the 2005 International Health Regulations (IHR), and highlighting priority pathogens, ‘system readiness’ and coordination requirements. The COVID-19 outbreak during 2020 has resulted in the further mobilisation of significant resources following this approach. As the World Health Organisation (WHO) Director-General put it in February, “We need to come together to fight a common enemy that does not respect borders, ensure that we have the resources necessary to bring this outbreak to an end and bring our best science to the forefront”“^ii^. Yet, as the COVID-19 pandemic has continued, multiple failures of response, globally and in diverse national and local settings, have emerged. Indeed, the very countries scoring the highest in 2019’s Global Health Security Index, the US and UK, have been among the most poorly-performing. The question this paper asks is whether such an approach responds effectively to the intersecting uncertainties evident in disease emergence and spread, whether the contextual social, political and cultural dimensions are sufficiently accounted for and what insights can be drawn for epidemics to come.

Preparedness frameworks have a long history. As Lakoff (2017) argues, concerns with ‘vital systems’ emerged in the Cold War era, creating a security apparatus, linked to a series of practices, devices and techniques, to prepare for military-nuclear threats. Within the health sector, the emergence of an explicit security framing, a departure from a concern with population-level public health (Lentzos and Rose 2009), accelerated during the SARS and avian influenza outbreaks in Asia in the early-2000s and laid the groundwork for epidemic preparedness. Preparedness is argued to be a new paradigm to manage infectious risk and outbreaks, aimed at creating a constant state of alertness and an ‘anticipatory imagination’ amongst policymakers (Lakoff 2015). In contrast to prevention, which aims to avert known risks, and precaution, which accepts that future events cannot be known but the worst outcome must be avoided, preparedness assumes that known events with unknown likelihoods can be responded to (Lakoff 2017). Thus, through processes of anticipation, an unknown future is invoked in the present, around which an array of preparedness approaches are galvanised (Adey and Anderson 2012).

Yet, despite the multitude of such efforts, as COVID-19 has starkly shown, the world remains unprepared (Caduff 2015). It is therefore time to ask who is being prepared for what, by whom and how is this done? What is missing from this mainstream approach?

In this paper, we rethink notions and applications of preparedness in several ways. First, we present a framework for thinking through the different dimensions of incertitude, examining how preparedness is constructed and how responses play out in relation to conditions of risk, uncertainty, ambiguity and ignorance. Second, through a series of cases – Ebola, Nipah, cholera and COVID-19 - we examine how preparedness plans unfold (or fail to), highlighting the diverse ways different actors navigate incertitude. Our chosen cases represent a range of disease types and kinds of transmission, from pathogens of supposedly zoonotic origin (Ebola, Nipah, COVID-19) to waterborne infections (cholera) and from diseases assumed to take a sudden, short outbreak form to those with longer-term dynamics - again cholera is a contrasting exemplar. The cases also focus on different places and contexts, from African and South Asian countries, conflict-affected settings in Yemen and Haiti, to the United Kingdom.

From these examples, we draw out some of the implications for thinking about uncertain futures in socially and culturally-grounded ways, and about preparedness as a social and political process within dynamic and uncertain situations, linked to diverse knowledges, cultures and practices. We argue that rethinking disease preparedness acts to collapse scales and connect global and local, expert and subaltern, formal and informal, present and future knowledges and practices. We suggest in conclusion that these insights offer pointers to approaches to preparedness that are more inclusive, just and ethical.

## Understanding incertitude

**Figure 1:**
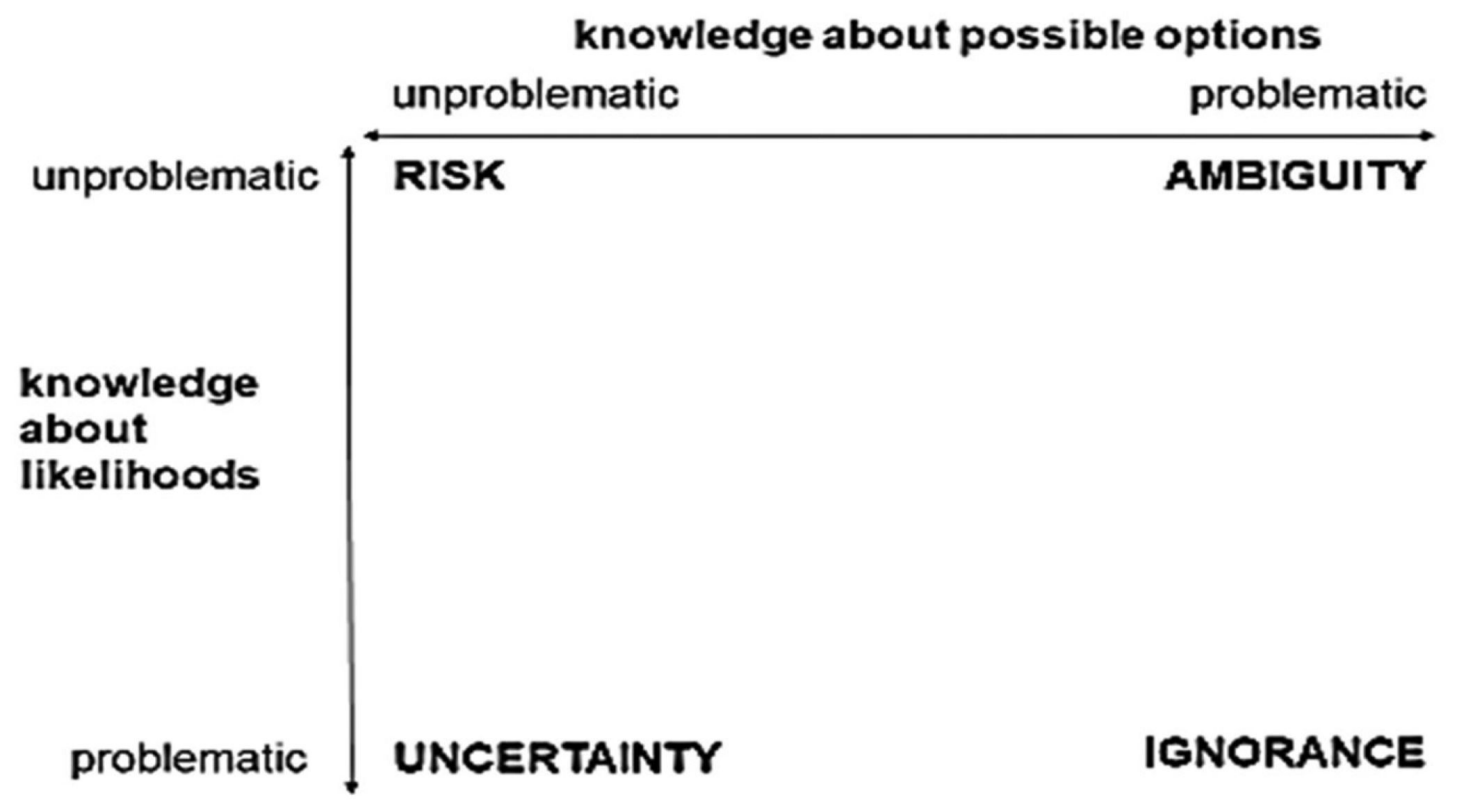
Four dimensions of incertitude (after Stirling, 1999)

In a conceptual framework for understanding the dimensions of ‘incertitude’ (Figure 1), Stirling (1999) contrasts risk, where the probabilities of both outcomes and their likelihoods are known (or deemed unproblematic), with uncertainty, where likelihoods are unknown; with ignorance, where outcomes and their probabilities are totally unknown and with ambiguity, where understandings and interpretations of potential outcomes are contested between actors.

We find that distinguishing between these types of incertitude enables a richer understanding of how disease dynamics are framed by different actors in different sites and over time. Thus, at different moments, disease dynamics can be constructed in terms of manageable risk, while at others only ignorance prevails, even if unacknowledged. As a disease and its social life unfold, ‘potential uncertainties’, constructed in future planning, may become actualised (Samimian-Darash 2013). Temporal shifts are both important for scientists and front-line practitioners, who must grapple in real-time with unfolding uncertainties, and for people experiencing outbreaks potentially alongside other vulnerabilities.

In exploring the four cases, the everyday lived experience of the four dimensions of incertitude, and the situated knowledge practices of those involved are examined. Located, ‘vernacular’ perspectives are contrasted with those constructed as part of global and national preparedness plans, and the tensions over different types of incertitude are examined. We reflect on Ebola, Nipah and cholera first, before asking what lessons can be drawn for COVID-19.

## Case 1: Ebola

Ebola has become an archetype for imaginaries of global pandemic threat and preparedness. Following the first recorded outbreak of this high-fatality viral haemorrhagic fever in 1976 in the Democratic Republic of Congo (DRC), a series of small outbreaks in Africa were managed through what became a standard risk-based approach, based on epidemiological modelling of spread, surveillance, isolation of cases, strict infection control, safe burial and contact tracing. The large 1995 outbreak in Kikwit, DRC catalysed a strengthening of preparedness planning approaches (Heymann et al., 1999). Meanwhile, scientific efforts focused on predicting risky ‘hotpots’: virus-hunting suggested outbreaks began with zoonotic spillover from a host in bats (Leroy et al., 2005), and ecological and climatic niche-modelling sought to predict when and where they would occur (Pinzon et al., 2004).

Despite decades of preparedness efforts, the 2013-2016 West African outbreak took the world by surprise, revealing deep uncertainties and ambiguities. Resulting in 11,323 reported deaths, it began in a region where Ebola in humans was previously unknown and spread fast amongst the dense, urbanised and highly-mobile populations of the Guinea, Sierra Leone and Liberia border area, amidst health systems and citizen-state relations rendered fragile by decades of conflict and mistrust. Initially ignored by the international community – based on assumptions that Ebola is ‘too deadly’ to generate large outbreaks – the social circumstances of the outbreak soon showed that complacency was unwise. By the time the epidemic was belatedly declared a ‘public health emergency of international concern’ in September 2014, it was already out of control, with subsequent modelling predicting millions of deaths.

Yet the linear assumptions and predictions of epidemiological models did not align with the region’s embedded entanglements of families, ethnic and linguistic affiliations and travel related to marriage visits, initiations and rural-urban trade, not easily recast as ‘risk factors’ for viral spread (Abramowitz 2017). Models also under-estimated learning amongst villagers and front-line workers, as they co-developed understandings of infection and adapted their practices around funerals, visiting and care (Richards 2016), thus reducing transmission so that (fortunately) the dire model predictions did not come true.

Ambiguities emerged in understandings of ‘Ebola’. Some local populations considered the disease and control efforts as a fabrication of foreign or governmental agencies, aimed at political control, genocide or land dispossession, sometimes responding with violence (Wilkinson and Fairhead 2017). Such fear and distrust reflected lived histories of inequality, conflict and intrusive foreign intervention amidst structural violence (Wilkinson and Leach 2014).

The biomedical, disease-focused response also clashed with villagers’ more socially and ecologically-integrated understandings of health and approaches to care. For villagers, bodily well-being, social life and livelihoods, and the productive and reproductive cycles of people, crops, animals and spirits are inextricably connected, and events that disrupt these relationships are catastrophic for society (Fairhead and Leach 1996). ‘Safe burials’ led by external teams, focused on biomedical infection control, often disrupted key aspects of care, visits and body preparation that villagers viewed as essential to ensure that the deceased becomes a good ancestor or to avoid societal disaster (Wilkinson et al., 2017). State and military-backed quarantines also exposed ambiguities between the priority of infection control and people’s food and livelihood needs (Hoffman 2016).

Following the West African epidemic, multiple international review panels documented ‘lessons learned’. Most mirror the standard preparedness ideas and plans of 20 years ago (Coltart et al., 2017), although with greater emphasis on community engagement, alongside an intensive focus on developing vaccines.

Refreshed plans were applied in the 2018-2020 Ebola outbreaks in North Kivu, yet control proved highly challenging. In a long-standing theatre of complex conflict, the response encountered myriad social and political uncertainties not amenable to containment through ‘community engagement’. Military action curtailed humanitarian access, armed groups made concerted attacks on Ebola Treatment Centres, and people prioritised immediate physical security over Ebola-related activities – leading to an ‘Ebola strike’ in October 2018. Furthermore, people’s historical political marginalisation generated distrust and plausible explanations of Ebola as a government plot. Fear and mistrust undermined both contact tracing and vaccination, with high levels of refusal. Congolese citizens equally wondered why Ebola was prioritised over other health issues in an already limited health system. In turn, mistrust was exacerbated by the disruptive ‘Ebola economy’; as resources and finance scaled up, so did contestation amongst already fragmented local political authorities. Thus, a new set of uncertainties and ambiguities unfolded in the dynamic relationship between local socio-politics and response activities.

Meanwhile, key areas of ignorance surround the longer-term dynamics of Ebola. Unknowns include the long-term implications of the disease in damage to organs and the nervous system and the extent of persistence of the virus in body fluids and so possibilities of sexual transmission. They also include the origin, spatiality and temporality of Ebola outbreaks. Predictive modelling assuming zoonotic spillover from bats continues (Redding et al., 2019), boosted by an origin narrative for the West African outbreak in which ‘patient zero’ was a toddler infected by a bat in the village of Meliandou, Guinea. Yet this dominant view is contested, including by villagers’ alternative narrative (Fairhead and Millimouno 2017). This relates the visit of a sick woman named Fanta from mining areas in Sierra Leone to a local healer, staying with (and infecting) the family of so-called ‘patient zero’. This alternative narrative gains support from research with Ebola ‘survivors’, revealing the possibility of flare-ups through reinfection. Thus Ebola dynamics might include viral persistence in human populations and more endemic forms, emerging into outbreaks amidst a myriad of interacting biological and social conditions - opening up further as yet unresolved uncertainties and possible surprises.

## Case 2: Nipah

Nipah virus was first identified as a novel pathogen two decades ago. Each successive outbreak in south and southeast Asia has catalysed further research, yet new uncertainties have continuously emerged. Indeed, unpredictability and change appear to be predictable characteristics of Nipah virus. Given this, and the high mortality associated with Nipah infection, the pressure on policy-makers and public health practitioners to foreground preparedness plans and ‘roadmaps’ for action is particularly intense. Systems for early detection, point-of-care diagnostics, rapid field epidemiology and access to therapeutics are central.

The emergence of Nipah virus in Malaysia in 1998 could be viewed as a classic ‘Disease X’ event. Initially, the cluster of neurological symptoms in pig farmers and abattoir workers was attributed to Japanese Encephalitis. However, this was disputed and investigation led to identification of the cause as a novel paramyxovirus (Chua et al., 2000). Ambiguities on the scientific front have thus been present from the outset. The epidemiological link for spillover was made to bats infecting pigs and then humans (*ibid*. 2000), while no human-to-human spread was detected.

The next outbreak occurred in 2001 but, surprisingly, in a geographically-remote site, in Bangladesh. The Bangladeshi strain is now known to be distinct: the disease was more aggressive, with human-to-human transmission and high mortality (Banerjee et al., 2019). Investigation of modes of transmission implicated bats, including the harvesting and drinking of raw palm sap, contaminated by bat secretions (Luby et al., 2006). The efforts of scientists in uncovering the complex transmission route within an ‘evidence-based’ paradigm did not match with local people’s understandings of fate and misfortune. Additional ambiguities concerned the importance of livelihoods derived from palm sap collection and also norms of care of the sick. Culturally-sensitive interventions, based on an appreciation of local beliefs and socio-economic realities, had greater success (Parveen et al., 2016).

By 2015, 17 further outbreaks had occurred in Bangladesh, with others in India and the Philippines, each with distinctive characteristics (Banerjee et al., 2019). Concerns have been raised about emergence and detection in geographic areas not previously high on ‘hotspot’ lists, especially as fruit bats are widespread in Africa and antibodies have been detected (Hayman et al., 2008). Thus, uncertainties have manifested in several ways - in unexpected geographic locations for outbreaks; in strains emerging with different degrees of virulence and various clinical syndromes and in different routes of transmission, thus implicating different animal hosts and human occupations. Diseases like Nipah will therefore inevitably create difficulties for accurate prediction.

A multi-pronged preparedness approach has been adopted by research and response agencies, with a marked emphasis on ecological surveillance and prediction, with intensive monitoring of bat populations potentially assisting in revealing new dynamics of viral shedding, enhancing the accuracy of predicting spillover events (Epstein et al., 2016). Thankfully, extensive human-to-human transmission has not occurred with Nipah, although mutations are possible, raising the spectre of significant epidemic spread.

The underlying assumption in the scientific community is that persistent scientific endeavour will ultimately resolve the ongoing uncertainties. Preparedness plans invoke on paper an ordered hierarchy and a standardised progression of steps, allowing a shift from uncertainty or ignorance to the domain of risk management in moments of outbreak crisis. A further strategy evident in the face of initial complete ignorance and then ongoing uncertainty, is to argue that robust systems of surveillance and preparedness are able to detect the unexpected and respond speedily; in other words to reveal the contours of uncertainty. Therefore, ‘roadmap’ discussions place considerable emphasis on acceleration of vaccine discovery, low-cost diagnostics and cutting-edge therapeutics, with R&D presented as the ultimate ‘silver bullet’ to defeat Nipah (WHO 2019) – if not at source, at least within humans.

Nosocomial transmission also exposes a hidden dimension of the Nipah case; lurking in the wings of the ‘outbreak’ drama is a reality that receives far less attention. This is the story about failures in basic hygiene, inadequate numbers of ambulances, lack of medical equipment, inadequate technology to detect and diagnose febrile illness and limited resources to provide medical support in the case of overwhelming illness. The development of a vaccine and immunoglobulin is considered as a priority in order to improve the clinical outcomes of the disease, amidst warnings of the potential for larger outbreaks in urban settings (Donaldson and Lucey 2018). Yet, whilst health systems in potential ‘hotspot’ areas remain weak and universal access to healthcare limited, the ability for case detection and laboratory identification is hampered^iii^. Basic public health provision and care, which would additionally address high morbidity and mortality across a range of other poverty-related illnesses, is likely to compete with investment in preparedness efforts for a Nipah, which remains rare and off-the-radar of ordinary people who experience other everyday adversities.

## Case 3: Cholera

Cholera is a devastating disease. Caused by the bacterium, *Vibrio cholerae*, and transmitted through contaminated water and food, there are between 1.3 and 4 million cases per year, across nearly 50 countries, resulting in 21,000 to 143,000 deaths, many of them children (Ali et al., 2015). Cholera’s impacts are concentrated in places that are poor and marginalised, often characterised as a disease of poverty, displacement and conflict. Here we reflect on Haiti in 2010 and Yemen from 2016.

In many respects cholera outbreaks are predictable, and certainly preventable and treatable, but a number of uncertainties combine. The El Tor cholera biotype has the possibility of asymptomatic carriers and then sudden, virulent spread (Mutreja et al., 2011). Symptoms are initially non-specific, and many live with diarrhoea in poor sanitation conditions anyway. Denial is a factor too, as admitting to a cholera outbreak may have major consequences for the economy. In contexts of war and conflict, infrastructural decay and poor health services, the possibilities of cholera outbreaks are always evident.

Cholera has generated an array of preparedness plans to guide outbreak responses (WHO 2017b). Attempts to tame uncertain futures are made through risk-based models - for example, linking cholera outbreaks to El Niño climatic events (Moore et al., 2017) and to algal blooms in rivers and estuaries (De Magny et al., 2008). Anticipatory preparedness practices include the surveillance of cases and laboratory testing, deployment of rapid response teams, creation of treatment centres, stockpiling and supply of oral rehydration kits and cholera oral vaccine, as well as requirements for sectoral coordination.

When cholera struck Haiti in October 2010, following a major earthquake, it was a total surprise. Haiti had not experienced cholera outbreaks before and the population was extremely vulnerable. Cholera quickly spread, as there were many displaced people and settlements had been destroyed. Even before the earthquake, in the capital Port-au-Prince, only half the population had access to latrines and a third to tap water (Farmer et al., 2011).

Initially, no-one knew where the disease had come from. Ignorance prevailed. Rumours and suspicions circulated, and disputes over the outbreak emerged. One explanation was environmental, arguing that climate and other factors had resulted in the sudden emergence of cholera. The other argued that an asymptomatic Nepalese UN peacekeeping soldier carried the disease and it had spread from the base due to poor sanitation. The latter theory, in the end, proved correct thanks to gene sequencing that traced the origins (Chin et al., 2011), but there was a long, tense period when no-one knew for sure.

This ambiguity resulted in major controversy. For many Haitians, the UN troops represented foreign interference, and local state officials and communities protested against them (Lemay-Hebert 2014). Cholera for them was a scourge that reflected marginalisation and exploitation by foreign powers. According to local narratives in rural areas, where the disease spread most, cholera was related to the ‘hidden sickness’ of underdevelopment and structural vulnerability (Koski-Karell et al., 2016). Addressing such questions of social and political marginalisation, however, is rarely part of preparedness plans.

Starting from September 2016, Yemen has suffered the largest-ever recorded cholera outbreak, in the midst of a vicious war. The preparedness plans that did exist were implemented unevenly (Spiegel et al., 2019). For example, treatment centres and oral rehydration corners had patchy coverage and cross-sectoral coordination was poor. Despite the establishment of a global stockpile in 2013, a vaccination programme only took off in mid-2017. Conflict has divided the country, and the rebel areas have had significantly higher cholera incidence, with less external support (Kennedy et al., 2017).

But would a preparedness plan have even been possible? There were so many uncertainties in a rapidly-changing setting of conflict and humanitarian crisis. Who could know where Saudi airstrikes would be targeted? Who could develop a plan for delivery in Houthi rebel-held areas under conditions of extreme insecurity? How can state-led sectoral coordination work in conditions with such minimal capacity? Coping in real-time was difficult enough, let alone providing a plan based on an unknown and unknowable future.

Once again, it is politics that generates vulnerability: those living outside government-controlled areas, and so subject to sustained bombing by Western-backed Saudi forces, have been far the most vulnerable. The uncertainties generated by regional and global geopolitics therefore have a direct impact on suffering. Again, preparedness plans, focused on technical risk management, never discuss such political issues, but they cannot be ignored.

Cholera has always been a political disease, rooted in inequality, often racialised (Briggs 2003) and generating protest and confrontation between citizens and authority, whether the state or the medical profession (Evans 1988). Even before the removal of London’s Broad Street pump-handle, riots across Europe in the 1830s showed how citizens saw cholera as representing more than just a deadly disease; a signifier of exploitation by the ruling classes and being disparaged by the professions (Cohn 2017). Echoes of such struggles around class, identity and rights are evident in recent outbreaks. With the disease so embedded in politics and constructions of citizenship – and so relationships with the state, science and ‘development’ (Chigudu 2020) - the idea of assembling technical plans for preparedness, based on the management of risk, without considering other dimensions of uncertainty, become absurd.

Unless preparedness practices – modelling, surveillance, stockpiling, administering treatments and so on - are deployed with a deeper understanding of context, with the real lives of those who are vulnerable to cholera at the centre, plans will fail; unravelling through a combination of inevitable ignorance, deep uncertainties about everything from the epidemiology to cultural behaviours and ambiguities over the origins of and reasons for outbreaks.

## Case 4: COVID-19

The ongoing COVID-19 pandemic has been a major test for pandemic preparedness at a global level. At the time of writing, 77 million cases had been confirmed globally, resulting in 1.69 million deaths. The toll in the UK, our case example here, has been high, with around 80,000 deaths and 3 million cases to date.

The UK pandemic response has largely relied on conventional risk-based epidemiological models to predict the spread of the disease. These models, coupled with mapping techniques, enable policy-makers to determine reproductive number (R0) values and identify areas ‘at risk’ in order to evaluate the potential effect of public health interventions. Such epidemiological modelling played an important role in sparking government policy on social distancing and quarantining to avoid overwhelming health facilities (Czyzewski 2020).

Yet we remain ignorant about many aspects of the disease, both in terms of its transmission and of its medical effects. Whilst respiratory transmission is the accepted main transmission mechanism, the relative weight of airborne or droplet transmission is debated (The Lancet Respiratory Medicine 2020). The precise role of asymptomatic (and pre-symptomatic) transmission, the immune reaction, the importance of reinfection, the role of genetic predisposition, as well as the possibility of seasonality in transmission are all examples where uncertainty and ignorance prevail. The long-term medical implications of COVID-19 are also still unclear. Vascular and neural damage is creating medium-term problems through chronic cases of ‘long COVID’ in which symptoms continue (Mahase 2020).

Putting such sources of deep uncertainty aside, the UK’s preparedness planning was initially based on expectations from pandemic influenza and resulted in the down-playing of investment in track-and-trace systems. Rather than acknowledging the uncertainties emerging from dealing with a novel virus and incorporating diverse scientific explanations, the UK government has claimed to ‘follow the science’, yet framed ‘the science’ narrowly through reliance on risk-based epidemiological modelling (Rhodes et al., 2020).

As in the case of Ebola, a lot of faith has been put in the production of a vaccine as a way of decreasing dependence on non-pharmaceutical interventions (physical distancing, lockdowns and travel bans) and test-trace-isolate mechanisms. At the time of writing, three vaccines have been approved in the UK and 64 vaccines are being tested in clinical trials on humans. Two of those vaccines are currently being administered to vulnerable populations across the country, although it remains uncertain whether vaccines will award long term protection or prevent asymptomatic transmission.

COVID-19 mirrors the cases described above, as uncertainties, ambiguities and sources of ignorance inevitably emerge. In the UK, socio-economic and geographical differences have resulted in contrasting exposure, severity of symptoms and mortality rates, confounding simplistic risk models. Class, race, age, occupation, living conditions (e.g. living in care homes) and other social dimensions have shaped the capacity to carry out physical distancing (e.g. through working remotely) and the likelihood of co-morbidities linked to disease severity (diabetes, heart disease, etc.) (PHE 2020).

The pandemic has unfolded in unexpected ways, again challenging simple models. Despite the government assuming that people would resist guidelines, events were cancelled and people chose to physically-distance well before the government-imposed lockdown. The knowledge of local public health officials and health workers also influenced the response, as did patterns of mutual aid and community support that emerged following imposed lockdowns.

The appearance of new and more contagious variants (identified in the UK and South Africa) has required a rethink of the tier system for the geographical implementation of physical distancing measures, and given rise to unexpected lockdowns. It is uncertain whether new variants can evade therapeutics or antibody responses, and whether approved vaccines will work against them (Mahase 2020).

As has occurred in other cases, tensions emerged between public health priorities and other goals. The UK government often framed the COVID-19 response as a trade-off between saving lives and protecting jobs and the economy. Lockdowns, transport bans and social-distancing measures have inevitably had a significant economic impact. Yet such indirect effects have also been distributed unequally. People in informal or precarious employment and people on low incomes have had to continue work in order to survive. Ambiguities also emerge between the goal of fighting COVID-19 and other health priorities. The central goal of containing COVID19 has had an impact on health service provision and access, likely resulting in significant increases in excess morbidity and mortality (Hrynick et al., 2020).

The longer-term dynamics of COVID-19 remain unknown. Non-technical solutions and a greater inclusion of plural knowledges in the response were pursued after the first wave, but the arrival of the vaccines has meant a reversal to technical solutions as ‘silver bullets’. Simple elimination with a vaccine, however, seems unlikely. Flare-ups are to be expected, and questions remain about how it might transform into an endemic disease, with mostly those from lower socio-economic backgrounds suffering its effects. The long-term dynamics remain unknown. Viral mutation, and the movement of people from countries that do not achieve herd immunity through vaccination will complicate elimination. The UK had invested significantly in pandemic preparedness planning, instead of incorporating responses from below the UK focused on different disease scenarios, and then relied on inevitably limited risk-based models to guide responses. The outcomes to date have been both devastating and discriminatory.

## Rethinking disease preparedness

**Figure 2:**
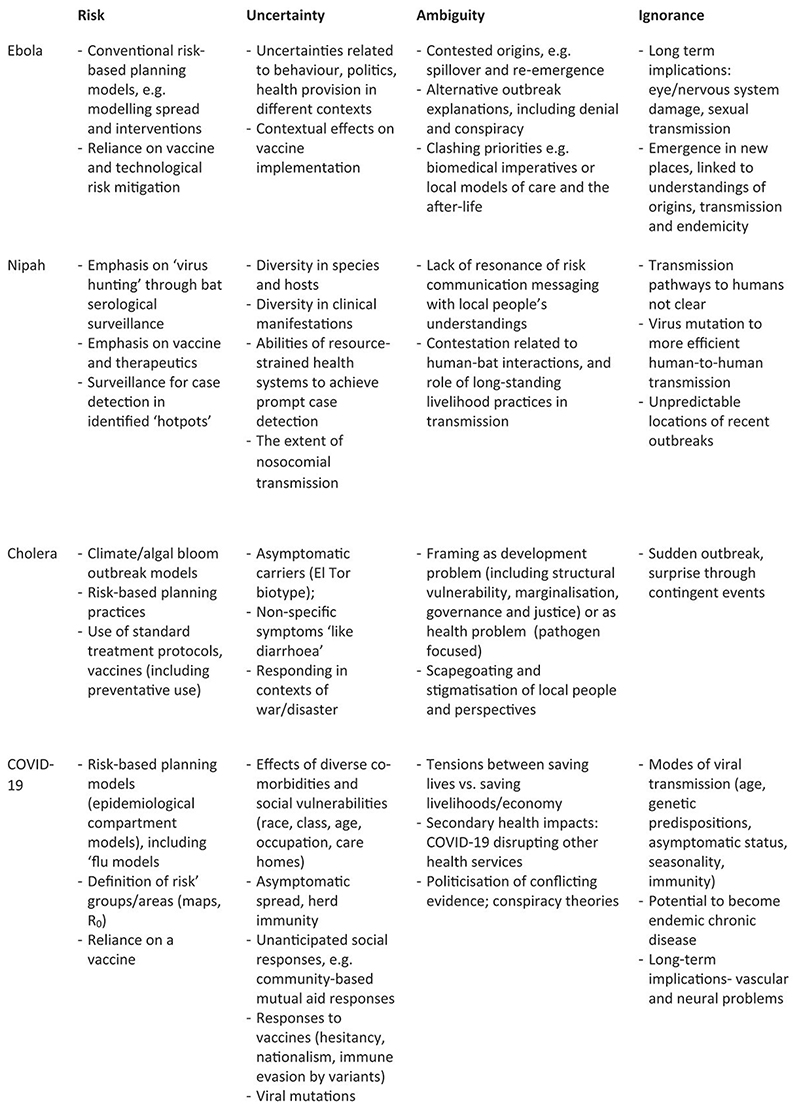
Dimensions of incertitude: four disease cases.

Current global debates rarely surface the important distinctions between different dimensions and experiences of incertitude. Indeed, much disease preparedness effort has focused on reducing uncertainty, ambiguity and ignorance to more manageable, calculable risks. These are then more amenable to established response efforts, whether through modelling and surveillance, virus hunting, ecological-niche modelling and investing in new therapeutics and laboratory research. Across the four cases, our framework identifies multiple uncertainties, ambiguities and sources of ignorance around the objects and goals of preparedness and how plans are enacted (or not) across different temporal, spatial and institutional scales (Figure 2).

Risk-focused strategies are important but not sufficient, and their dominance – and reassuring allure - can mean other considerations are overlooked. Looking beyond risk, or what is assumed to be risk, we repeatedly see that the diversity of contexts, pathogens and people shape how an outbreak unfolds in unpredictable ways. Social-political contexts, which include war, protracted crises and longer histories of structural violence, are sources of new or ongoing uncertainties that standardised plans and protocols are ill-adapted to. The cases further emphasise the disjunctures between understandings of scientists, practitioners and policy-makers and those living in diverse local settings. Ambiguities are revealed in scientific contestation, but also centre on diverse knowledges, interpretations and values around the character and significance of a particular disease and the origins of a potential outbreak. Ambiguities also revolve around how disease response is balanced against other health and livelihood priorities, as well as how global health security is framed against local freedoms, ethics and justice.

What, then, does this imply for rethinking disease preparedness at this critical juncture when COVID-19 prompts urgent renewed reflection? In each case, the failures of standard risk-based approaches to preparedness are clear. Political, social and cultural factors impinge to unravel the neat plans and protocols. But what might replace the standard, failing approach? The cases point to four important cross-cutting principles applicable to all, which we describe and then briefly illustrate with one practical example in each instance of how these principles might be applied.

### Knowledge

In the unfolding of uncertainties on the ground, there is a continuous interaction of different types of knowledge. Yet, a dominant, expert-driven, risk-based, centralised style of knowledge construction is at play, often removed from local settings where diseases are experienced, frequently precluding the meaningful engagement with contextually-grounded, alternative knowledges. As our cases underscore, who holds knowledge about what in a particular setting becomes pertinent in thinking about preparedness. Understanding how knowledge is constructed, presented and held in particular social and cultural contexts of course also challenges a linear, expert view of risk and uncertainty, and emphasises instead the indeterminacy and contingency of day-to-day experience (Wynne 1996).

As seen in all cases, the everyday, embodied, experiential knowledge, practice and learning by those affected is often ignored. Rethinking preparedness therefore means an openness to a greater plurality of knowledges – how evidence is assembled, how it is interpreted and by whom. This requires an acceptance that multiple knowledges will collide and this in turn requires iterative learning, dialogue and collaboration. We must appreciate that models (epidemiological or otherwise) are always partial ways of presenting a story, and that to address deeper uncertainties requires triangulations amongst multiple ‘modelling’ approaches (Scoones et al., 2017). This requires a democratisation of knowledge-making in learning processes among actors involved in modelling and field responses over time. The Nipah case shows instances where researchers engaged with villagers in Bangladesh, taking seriously their aetiological understandings of the new illness and scepticism about scientific explanations, and adjusted interventions accordingly.

### Time and space

Mainstream approaches to preparedness are constructed in terms of a defined, anticipated future event, around which risk-management approaches are constructed. All our cases however reveal the limitations of this approach. There are deep ambiguities about stories of origin, onset and conclusion of a disease event; and indeed whether it is an event at all or part of an on-going, everyday flow of disease dynamics, conditioned by structural, political-economic factors (Anderson et al., 2019).

Preparedness systems centre on the anticipation of an open future, where particular, defined, but uncertain, punctuated events might occur in certain ‘hotspots’. Socio-technical imaginaries of anticipated future catastrophic disease events are invoked in the present through preparedness plans, models and practices, crystallising particular assumptions that then shape emergency interventions and responses (Adey and Anderson 2012). In addition to the calculation of risk through predictive models, the construction of imaginaries of preparedness involves a number of other spatial and temporal moves. One is to generate hierarchies of control, distancing the coordination and response teams, the research laboratories and the centralised stockpiles and early-warning facilities from the complex, uncertain particularities of disease environments, pathogens and people. Another is to specify preparedness in terms of an event or a series of defined stages leading up to a disease emergency and streamlining the response (Abeysinghe 2013).

Yet, in practice, our cases reveal interlocking, collapsed and folded temporalities, in which on-going biological, social and political dynamics interplay with each other over different time-scales. Future uncertainties are not necessarily apprehended as linear and ordered, but collapsed and layered (Bear 2016). A modernist vision of progress, and so liberal conceptions of the future, is thus challenged and with this the prospect of controlling singular, manageable futures through a risk-based calculus (Scoones and Stirling 2020).

The current architecture of disease preparedness is however premised on the delivery of interventions around a specified event, but our cases suggest a more continuous, adaptive, connected set of actions over time and space. For example, in the case of cholera, a focus on the lived-with experience of those suffering the disease, rather than simply mobilisation around an outbreak event, would highlight how people understand, cope with and respond to a range of gastrointestinal conditions continuously. Responses to cholera outbreaks could thus be attuned to grounded, everyday experiences, where in certain places disease impacts and social and political marginalisation emerge together over time.

### Institutions and administration

Administrative systems are co-constructed with systems of risk management and control and, as we have seen across the cases, these are unable to deal with deep uncertainties and ongoing ambiguities. The cycle of ‘lessons learned’ assessments after each outbreak acts to reinforce and reproduce managerial control, while feeding into a narrative of ‘progressive improvement’ by outside, usually Western, agencies imposed on other people and places.

Targets, audits, standard operating procedures, protocols and frameworks satisfy the needs of bureaucracies, but obscure uncertainties, ambiguities and sources of ignorance.

Idealised preparedness systems rely heavily on assuming a functioning local infrastructure. As Farmer (2014) argues, stopping infection diseases requires ‘staff, space, stuff and systems’. This means mobilising skills, practices and materials that allow public health, clinical care and water and sanitation systems to function effectively, but crucially also means collaboration across actors with diverse knowledges and imaginaries, building trust and solidarity.

Our cases show how the distinctions between ‘global’ and ‘local’ settings are in practice collapsed, and the hierarchy of institutional responsibilities, so important to maintaining the illusion of risk-based control, is constantly subverted. Rather than implementing a pre-defined framework, flexible skills for responding to uncertainties and generating reliability are required (Roe 2013). Reflection on the effects of ‘one-size-fits-all’ responses to the COVID-19 pandemic highlights the need to devise means for more flexible, localised responses. For example, if the UK COVID-19 response had relied from the onset on local institutions, civil society and local authorities rather than top-down management, it might have achieved better outcomes, such as through effective track-and-trace systems.

### Ethics and justice

As our cases show, a focus on ambiguities shows that preparedness means very different things to different people. Preparedness that leads to the rolling-out of standard response mechanisms - such as quarantining, safe-burials, animal culling and so on – can generate injustices. They can run against deeply held priorities, moral economies of care and kin relations; even the material means of survival. This suggests the need for a more open and transparent deliberation about priorities, striking difficult balances between public health notions of control, individual and collective rights, ethics and social justice. Sometimes this may lead to mutually-acceptable solutions; sometimes there is greater contestation. This raises vital questions about how to ensure proportionate and just public health responses for future epidemics.

Given the deep historical, structural inequalities and forms of violence embedded in many settings where preparedness interventions take place, there is a danger that supporting local knowledge and practices of ‘coping’ and ‘resilience’, will simply reinforce historical injustices (Duffield 2011). A new ethics of preparedness needs to acknowledge histories, political economies, unequal relationships and everyday injustices. Seeking to build collective solidarities that confront power and hierarchy thus means going beyond person-centred dignity and engagement.^iv^ The example of Ebola in West Africa provides a major insight here. As much as local learning is highlighted as a key factor in bringing the epidemic under control, an equally important process was that the authorities in charge of the response began to take local people’s priorities - be they around burials or care-giving – and their long-held fears and suspicions of outsiders seriously and respectfully. Control interventions only became effective when they began to blend biomedical concerns with social and moral imperatives.

## Conclusion

These four themes suggest a very different vision for disease preparedness thinking. Together they require moving beyond resource investment and system strengthening to rethink preparedness more fundamentally as a dynamic social, cultural and political process.

This requires accepting the limits of expert-led risk management - and so reliance on models, predictions and control-oriented solutions. It means seeking out alternative, open-ended methodologies that embrace uncertainty, while acknowledging that ignorance exists and surprises will occur. It requires socially-just and ethical preparedness plans to emerge through dialogue and deliberation, co-constructing both new knowledges and social orders for preparedness (Jasanoff 2004). And it requires addressing ambiguities head-on through the opening up and navigation of social and political contestations over time, reflecting on different disease narratives, frames and logics, from multiple perspectives.

COVID-19 has added urgency to our call for a fundamental rethinking of preparedness approaches and the need for a significant departure, rather than simply requests for greater investment to do more of the same. The lessons from past experiences offer important ways forward.

## Footnotes

ii WHO R&D Roadmap meeting 12-13 Feb 2020, https://www.who.int/news-room/detail/12-02-2020-world-experts-and-funders-set-priorities-for-covid-19-research

iii
https://www.healthsystemsglobal.org/blog/364/Health-systems-must-go-beyond-sick-care-for-Universal-Health-Coverage.html

iv
https://www.nuffieldbioethics.org/assets/pdfs/RGHEfull_report1.pdf
